# Following Glucose Oxidase Activity by Chemiluminescence and Chemiluminescence Resonance Energy Transfer (CRET) Processes Involving Enzyme-DNAzyme Conjugates

**DOI:** 10.3390/s111110388

**Published:** 2011-10-31

**Authors:** Angelica Niazov, Ronit Freeman, Julia Girsh, Itamar Willner

**Affiliations:** Institute of Chemistry, Center for Nanoscience and Nanotechnology, The Hebrew University of Jerusalem, Jerusalem 91904, Israel

**Keywords:** sensor, glucose, chemiluminescence, DNAzyme, quantum dots, hydrogen peroxide, G-quadruplex, chemiluminescence resonance energy transfer

## Abstract

A hybrid consisting of glucose oxidase-functionalized with hemin/G-quadruplex units is used for the chemiluminescence detection of glucose. The glucose oxidase-mediated oxidation of glucose yields gluconic acid and H_2_O_2_. The latter in the presence of luminol acts as substrate for the hemin/G-quadruplex-catalyzed generation of chemiluminescence. The glucose oxidase/hemin G-quadruplex hybrid was immobilized on CdSe/ZnS quantum dots (QDs). The light generated by the hybrid, in the presence of glucose, activated a chemiluminescence resonance energy transfer process to the QDs, resulting in the luminescence of the QDs. The intensities of the luminescence of the QDs at different concentrations of glucose provided an optical means to detect glucose.

## 1. Introduction

The hemin/G-quadruplex horseradish peroxidase (HRP)-mimicking DNAzyme attracts substantial research effort as catalytic [1] or electrocatalytic label [2,3] for the amplification of biorecognition events. The HRP-mimicking DNAzyme has been used as a catalyst for the colorimetric or chemiluminescence detection of DNA [4–6], aptamer substrate complexes [7–10], and for the analysis of metal ions [11–15]. The hemin/G-quadruplex nanostructure has also been implemented as an amplifying optical label for biorecognition events using surface plasmon resonance (SPR) spectroscopy [16], and as an electrocatalyst for the amplified electrochemical detection of DNA [17], aptamer-substrate complexes [18], and metal ions [19]. Recently, semiconductor quantum dots (QDs) have attracted substantial research interest due to their unique optical features and particularly, their size-controlled luminescence properties [20]. Numerous studies have used QDs for developing fluorescence/fluorescence resonance energy transfer and photoelectrochemical-based biosensors, and the advances in the field have been extensively reviewed [21–24]. The hemin/G-quadruplex nanostructure was found to act as an electron transfer quencher of the luminescence of the quantum dots, and the system was used to sense DNA or aptamer-substrate complexes [25], or to follow the telomerization process [26]. Also, the hemin/G-quadruplex was conjugated to quantum dots and used to develop chemiluminescence resonance energy transfer (CRET)-based DNA sensors [27] or aptasensors [28]. The DNAzyme-generated chemiluminescence by these systems, in the presence of H_2_O_2_/luminol, provided the energy needed to excite the quantum dots. This enabled the development of QDs-based chemiluminescent DNA or aptamer-substrate sensors with no external illumination. Also, by using different sized QDs, the multiplexed analysis of DNAs by this analytical platform was demonstrated [27].

In the present study we describe the coupling of the HRP-mimicking DNAzyme to the catalytic protein, Glucose Oxidase (GOx), to generate glucose oxidase/peroxidase-mimicking DNAzyme conjugates. The biocatalytic functions of the GOx enzyme activate an enzyme cascade that leads to the generation of chemiluminescence. We also demonstrate that the chemiluminescence, generated by the HRP-mimicking DNAzyme, in the presence of luminol and the GOx-generated H_2_O_2_, stimulate the chemiluminescence resonance energy transfer (CRET) to CdSe/ZnS QDs and triggers on the luminescence of the QDs. This process was used to probe the catalytic activity of glucose oxidase.

## 2. Experimental Section

### 2.1. Chemicals

The following nucleic acids were purchased from Sigma Genosys:

1. 5′-NH_2_-(CH_2_)_6_-TTTTTTGGGTAGGGCGGGTTGGG-3′.
2. 5′- HS- (CH_2_)_6_-TTTTTTGGGTAGGGCGGGTTGGG-3′.

The bifunctional cross-linkers bis(sulfosuccinimidyl)suberate (BS^3^) and *N*-(ε-maleimidocaproyloxy) sulfosuccinimide ester (EMCS), were purchased from Pierce. Hemin was purchased from Frontier Scientific and was dissolved in DMSO without further purification. All other chemicals, such as glucose oxidase (GOx, EC 1.1.3.4 from *Aspergillus niger*), luminol, d-(+)-glucose, phosphate buffer (PB) and 4-(2-hydroxyethyl)piperazine-1-ethanesulfonic acid sodium salt (HEPES) were purchased from Sigma Aldrich and used as supplied. Ultrapure water from a NANOpure Diamond (Barnstead) source was used in all of the experiments.

### 2.2. Synthesis of the GOx–DNAzyme Conjugate

The DNAzyme oligonucleotide **1** was dissolved in phosphate buffer (10 mM, pH 7). GOx was dissolved in HEPES buffer (25 mM, pH 7.4). A molar excess of the cross-linker, BS^3^, was added. The resulting solution was incubated for 20 min in room temperature and the excess of the linker was removed by a MicroSpin G-25 column. The resulting solution was reacted with **1** for 2 h. The excess oligonucleotide was removed from the solution by centrifugation (Amicon Ultra, 50,000 MWCO, Millipore) and the resulting **1**-GOx hybrid was diluted in HEPES buffer (25 mM, pH 7.4) containing 20 mM KNO_3_, 200 mM NaNO_3_.

### 2.3. Modification of the CdSe/ZnS QDs with the GOx–DNAzyme Conjugate

The GSH-capped QDs (3 nmol) in HEPES buffer (100 μL), were reacted with an excess of BS^3^, and the mixture was shaken for 20 min. The QDs were purified by precipitation by the addition of 0.5 mL of methanol to remove the excess of BS^3^. The QDs were re-dissolved in 25 mM HEPES buffer pH 7.4 containing the GOx enzyme (200 nmol) and the mixture was shaken for 2 h. The nucleic acid **2** was reduced by DTT and purified by a MicroSpin G-25 column. The freshly reduced **2** was reacted with an excess of EMCS for 20 min and was purified by a MicroSpin G-25 column. The resulting GOx-modified QDs were purified by one precipitation step and reacted with the EMCS-modified **2** for 2 h. Finally, the excess DNA was removed by precipitation of the QDs, and the purified particles were dissolved in HEPES buffer solution (25 mM, pH 7.4).

### 2.4. Determination of the DNAzyme/GOx Ratio

The loading of the enzyme with the nucleic acid was determined spectroscopically, by analyzing the residual non-bound DNA in the modifying solution. Knowing the concentration of the GOx enzyme, the DNAzyme/GOx ratio was calculated.

### 2.5. Determination of the Loading of the CdSe/ZnS QDs with the GOx-DNAzyme Hybrid

The absorption spectrum of the CdSe/ZnS nanoparticles of known concentration was recorded prior to the modification of the particles. The absorption spectrum of the GOx-functionalized CdSe/ZnS QDs was, then, recorded and the spectrum was normalized to the same OD value at 600 nm, observed for the non-modified QDs. Since GOx is not absorbing at 600 nm, the subtraction of the spectrum of the GOx-functionalized QDs from the non-modified QDs yielded the absorbance of the enzyme associated with the QDs. The absorbance difference at λ = 280 nm allows the calculation of the concentration of the GOx enzyme. Knowing the concentration of the QDs, the GOx/QDs ratio was, then, calculated. Afterwards, the loading of the DNAzyme on the GOx was calculated by analyzing the residual non-bound DNA in the modifying solution.

### 2.6. Chemiluminescent Analysis of Glucose by the GOx–DNAzyme Conjugates

For the chemiluminescent detection of glucose, a 5 μM solution of GOx-DNAzyme hybrid was added to a cuvette that included hemin (1 μM) and buffer solution (25 mM HEPES, 20 mM KNO_3_, 200 mM NaNO_3_, pH 7.4), the resulting solution was incubated for 15 min. A further incubation of 5 min was executed with different concentrations of glucose. Luminol (0.5 mM) dissolved in a buffer solution (25 mM HEPES, 20 mM KNO_3_, 200 mM NaNO_3_, pH 9) was added to the reaction mixture. Light emission at λ = 420 nm was measured using a photon counting spectrometer (Edinburgh Instruments FLS 920) equipped with a cooled photomultiplier detection system, connected to a computer (F900 v. 6.3 software).

### 2.7. The Analysis of Glucose by the GOx-DNAzyme Hybrid-Modified QDs Using CRET as the Readout Signal

A solution containing 1 μM hemin and GOx-DNAzyme modified QDs (1.5 μM) in 25 mM HEPES, 20 mM KNO_3_, and 200 mM NaNO_3_, pH 7.4 was incubated for 15 min. Subsequently, the solution was incubated with different concentrations of glucose for another 15 min. After adding 5 mM luminol, light emission intensity was measured by a photon counting spectrometer (Edinburgh Instruments, FLS 920) equipped with a cooled photomultiplier detection system connected to a computer (F900 v.6.3 software).

## 3. Results and Discussion

**Figure 1.**
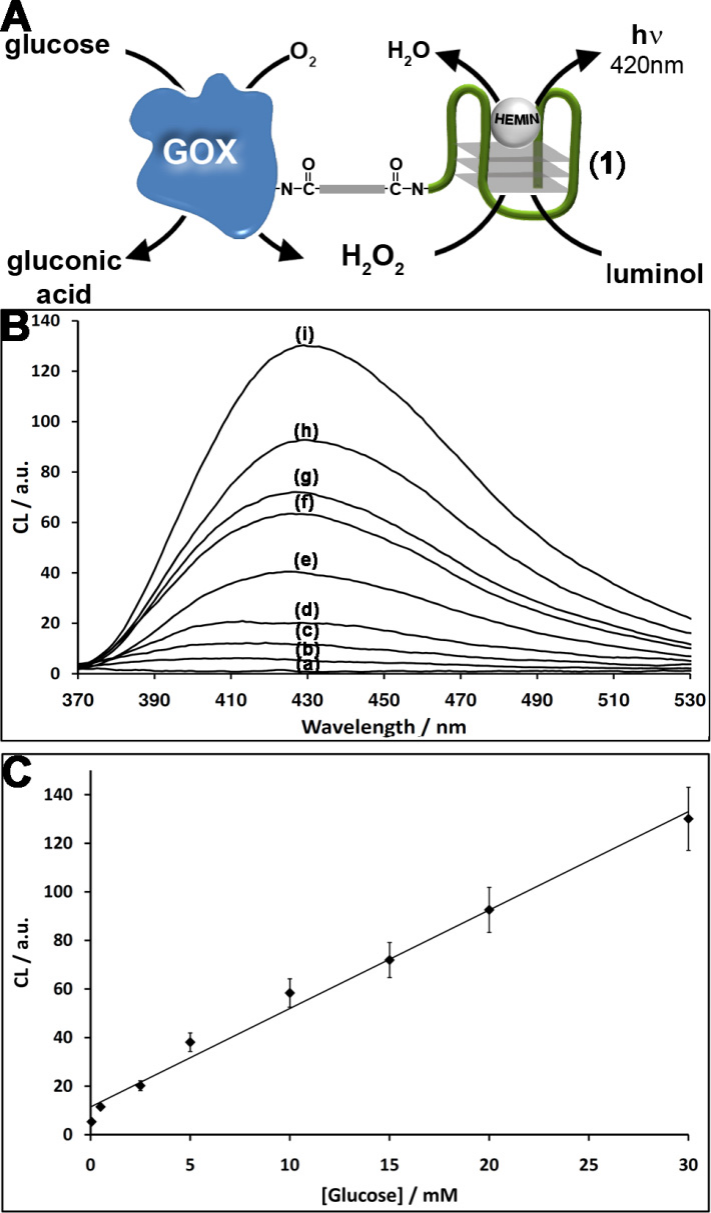
**(A)** Chemiluminescence analysis of glucose by DNAzyme-tethered glucose oxidase; **(B)** Chemiluminescence light intensities generated by the (**1**)-modified GOx in the presence of different concentrations of glucose: (a) 0 M (b) 0.05 mM (c) 0.5 mM (d) 2.5 mM (e) 5 mM (f) 10 mM (g) 15 mM (h) 20 mM (i) 30 mM; **(C)** Resulting calibration curve derived from the increase in the chemiluminescence signal at λ = 430 nm. Each data point is the average of N = 3 experiments. The error bars represent the standard deviation.

**Figure 2.**
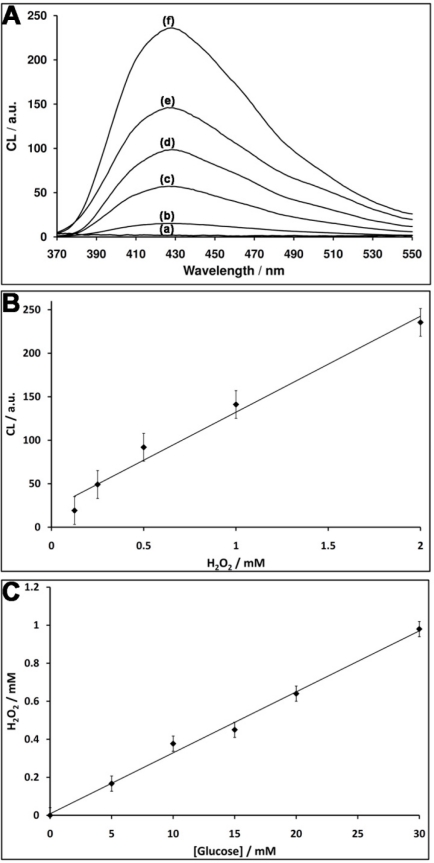
**(A)** Chemiluminescence light intensities generated by the (**1**)-modified GOx in the presence of different concentrations of H_2_O_2_: (a) 0 M (b) 0.125 mM (c) 0.25 mM (d) 0.5 mM (e) 1 mM (f) 2 mM; **(B)** Resulting calibration curve derived from the increase in the chemiluminescence signal at λ = 430 nm. Each data point is the average of N = 3 experiments. The error bars represent the standard deviation; **(C)** Calibration curve corresponding to the concentration of H_2_O_2_ generated by various concentrations of glucose.

The nucleic acid **1** consists of the G-rich HRP-mimicking DNAzyme, was tethered to glucose oxidase (GOx) [Figure 1(A)]. The GOx was first reacted with bis(sulfosuccinimidyl)suberate (BS^3^), followed by the covalent tethering of the amino nucleic acid **1**. The loading of the enzyme with **1** was determined spectroscopically to be *ca.* three DNAzyme units per protein. The DNAzyme-functionalized GOx conjugate was employed as a catalytic label to analyze glucose (Figure 1). The GOx-mediated oxidation of glucose to gluconic acid by O_2_ yields H_2_O_2_. The resulting H_2_O_2_ was then analyzed by the Hemin/G-quadruplex DNAzyme units by the generation of chemiluminescence at λ = 420 nm, in the presence of luminol. As the concentration of glucose controls the concentration of the resulting H_2_O_2_, the intensity of the emitted chemiluminescent light provides a quantitive measure for the optical analysis of glucose. Figure 1(B) shows the chemiluminescence intensities generated by the system in the presence of different concentrations of glucose. As the concentration of glucose increases, the content of the GOx-generated H_2_O_2_ increases, resulting in higher emitted chemiluminescence light. Control experiments revealed that no light was generated by the system upon the exclusion of either glucose, hemin or luminol. Also, no light was generated by the native, non-modified GOx in the presence of glucose, luminol and diffusional hemin. These control experiments confirm that the hemin/DNAzyme-modified GOx hybrid is essential for the generation of chemiluminescence. Figure 1(C) depicts the resulting calibration curve corresponding to the light intensities generated by different concentrations of glucose. The successful generation of chemiluminescence by the GOx/hemin/G-quadruplex hybrid is attributed to the GOx-mediated oxidation of glucose that yields a high local concentration of H_2_O_2_, close to the hemin/G-quadruplex DNAzyme active site, leading to the effective oxidation of luminol. To quantify the content of H_2_O_2_ consumed by the hemin/DNAzyme under the experimental conditions of the assay (5 min incubation), induced by different concentrations of glucose, we followed the chemiluminescence signal generated by the (**1**)-GOx hybrid in the presence of known concentrations of H_2_O_2_ (in the absence of glucose). Figure 2(A) shows the chemiluminescence intensities upon activating the oxidation of luminol by various concentrations of H_2_O_2_ in the presence of the hemin-G-quadruplexfunctionalized GOx. Assuming that the chemiluminescence generated by the oxidation of luminol relates directly to the concentration of the H_2_O_2_, the resulting calibration curve [Figure 2(B)], reflects the chemiluminescence intensities generated by different concentrations of H_2_O_2_ in the presence of the GOx/hemin/G-quadruplex hybrid. Using this calibration curve, we derived the concentration of the GOx-generated H_2_O_2_ generated in the presence of various concentrations of glucose [Figure 2(C)]. For example, in the presence of glucose concentration corresponding to 10 mM, we estimate that the concentration of H_2_O_2_ consumed by the hemin-G-quadruplex DNAzyme corresponds to 0.4 mM.

**Figure 3.**
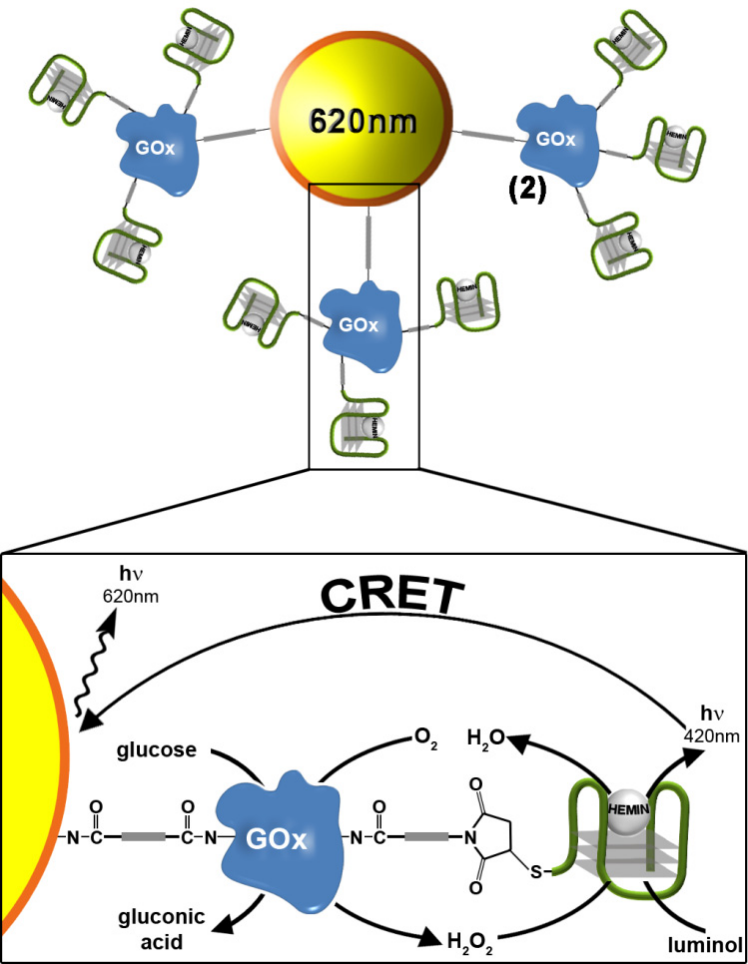
Analysis of glucose by the hemin/G-quadruplex-modified GOx that catalyzes the oxidation of glucose by oxygen and the formation of H_2_O_2_. The subsequent catalyzed generation of chemiluminescence in the presence of luminol/H_2_O_2_, results in the CRET process to the QDs.

We then examined the possibility of coupling the DNAzyme-GOx conjugate to CdSe/ZnS QDs with a goal that the generated chemiluminescence light will stimulate a chemiluminescence resonance energy transfer (CRET) process to the QDs and will trigger-on the luminescence of the QDs. Figure 3 illustrates the schematic configuration of the CRET system. The CdSe/ZnS QDs (λ_em_ = 620 nm) were modified with a glutathione (GSH) capping layer, and the enzyme glucose oxidase, GOx was covalently linked to the QDs. The spectra of the QDs were recorded before and after modification with GOx, and the loading of the QDs with the GOx enzyme was determined from the difference spectrum. Then, nucleic acid **2** was covalently tethered to the GOx-functionalized quantum dots. The loading of the GOx-modified QDs with the DNAzyme units was determined by analyzing the residual, non-bound DNA in the modifying solution. The spectroscopic analysis of the modified QDs indicated that approximately two hybrid units were associated with each particle (for the synthesis and characterization of the modified QDs, see the Experimental section). In the presence of hemin, luminol and glucose, the HRP-mimicking DNAzyme catalyzed the oxidation of luminol by the GOx-generated H_2_O_2_, resulting in the CRET between luminol and the QDs bioconjugates, as shown in Figure 3. This enabled the use of the QDs conjugate as luminescent reporter units to follow biocatalytic cascades and for the analysis of glucose.

**Figure 4.**
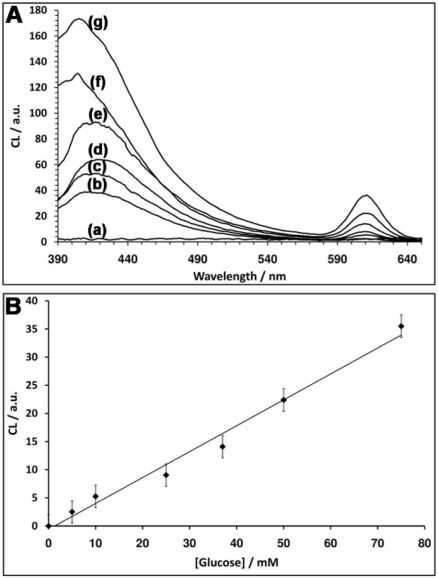
**(A)** Luminescence spectrum corresponding to the CRET signal of the QDs at λ = 610 nm in the absence of glucose, curve (a), and in the presence of different concentrations of glucose: (b) 5 mM (c) 10 mM (d) 25 mM (e) 37 mM (f) 50 mM (g) 75 mM; **(B)** Calibration curve corresponding to the increase in the CRET signal at λ = 610 nm. Each data point is the average of N = 3 individual measurements. The error bars indicate the standard deviation.

Figure 4(A) depicts the CRET signals of the QDs stimulated by different concentrations of glucose. As the concentration of glucose is higher, the CRET signals are higher, consistent with a higher content of GOx-generated H_2_O_2_. Figure 4(B) depicts the resulting calibration curve corresponding to the CRET signals triggered by different concentrations of glucose. The detection limit for the analysis of glucose corresponds to 5 mM.

## 4. Conclusions

The present study has described the use of DNAzyme-functionalized proteins as active conjugates that can follow biocatalytic transformations. Specifically, we modified glucose oxidase with the hemin/G-quadruplex horseradish peroxidase-mimicking DNAzyme, and generated a bifunctional catalytic hybrid for the optical detection of glucose. The hybrid activated a catalytic cascade where the glucose oxidase catalyzed the oxidation of glucose by oxygen to form gluconic acid and H_2_O_2_ that triggered-on the DNAzyme-catalyzed generation of chemiluminescence in the presence of luminol. Also, the coupling of the DNAzyme-modified GOx conjugates to CdSe/ZnS QDs enabled to follow the biocatalyzed oxidation of glucose via the CRET process to the QDs. This might be implemented to probe other oxidases that generate H_2_O_2_. Moreover, realizing that different sized QDs may be excited by the emitted chemiluminescence generated by luminol, this platform can be implemented for the multiplexed analysis of different oxidases and their substrates. One of the advantages of using CRET as a readout signal is the zero background signal in absence of glucose. Many of the electrochemical glucose sensors devices suffer from the perturbation of the electrical readout signal due to the non-specific oxidation of interferants, such as uric acid or ascorbic acid. The specificity of the CRET-based sensor to the GOx-mediated oxidation of glucose leads to a selective sensor for the target glucose substrate. Furthermore, one may realize that the resulting linear calibration curve (detection limit 5 mM) is in the range interesting for the analysis of glucose in diabetic patients. This suggests that it would be possible to design CRET-based sensors using a single point calibration process. Although the CRET-based glucose sensor reveals important features for future medical diagnostics, its practical application needs further optimization.

## References

[1] Willner I., Shlyahovsky B., Zayats M., Willner B. (2008). DNAzymes for sensing, nanobiotechnology and logic gate applications. Chem. Soc. Rev.

[2] Pelossof G., Tel-Vered R., Elbaz J., Willner I. (2010). Amplified biosensing using the horseradish peroxidase-mimicking DNAzyme as an electrocatalyst. Anal. Chem.

[3] Shen B.J., Wang Q., Zhu D., Luo J.J., Cheng G.F., He P.A., Fang Y.Z. (2010). G-quadruplex-based DNAzymes aptasensor for the amplified electrochemical detection of thrombin. Electroanalysis.

[4] Bi S., Zhang J.L., Zhang S.S. (2010). Ultrasensitive and selective DNA detection based on nicking endonuclease assisted signal amplification and its application in cancer cell detection. Chem. Commun.

[5] Xiao Y., Pavlov V., Gill R., Bourenko T., Willner I. (2004). Lighting up biochemiluminescence by the surface self-assembly of DNA-hemin complexes. ChemBioChem.

[6] Niazov T., Pavlov V., Xiao Y., Gill R., Willner I. (2004). DNAzyme-functionalized au nanoparticles for the amplified detection of DNA or telomerase activity. Nano Lett.

[7] Li T., Wang E.K., Dong S.J. (2008). G-quadruplex-based DNAzyme for facile colorimetric detection of thrombin. Chem. Commun.

[8] Li D., Shlyahovsky B., Elbaz J., Willner I. (2007). Amplified analysis of low-molecular-weight substrates or proteins by the self-assembly of DNAzyme-aptamer conjugates. J. Am. Chem. Soc.

[9] Li T., Wang E., Dong S.J. (2008). Chemiluminescence thrombin aptasensor using high-activity DNAzyme as catalytic label. Chem. Commun.

[10] Shlyahovsky B., Li D., Katz E., Willner I. (2007). Proteins modified with DNAzymes or aptamers act as biosensors or biosensor labels. Biosens. Bioelectron.

[11] Li T., Shi L.L., Wang E.K., Dong S.J. (2009). Silver-ion-mediated DNAzyme switch for the ultrasensitive and selective colorimetric detection of aqueous Ag^+^ and cysteine. Chem.-Eur. J.

[12] Li D., Wieckowska A., Willner I. (2008). Optical analysis of Hg^2+^ ions by oligonucleotide-gold-nanoparticle hybrids and DNA-based machines. Angew. Chem. Int. Ed.

[13] Li T., Wang E., Dong S.J. (2009). G-Quadruplex-based DNAzyme as a sensing platform for ultrasensitive colorimetric potassium detection. Chem. Commun.

[14] Li T., Li B.L., Wang E.K., Dong S.J. (2009). G-quadruplex-based DNAzyme for sensitive mercury detection with the naked eye. Chem. Commun.

[15] Elbaz J., Shlyahovsky B., Willner I. (2008). A DNAzyme cascade for the amplified detection of Pb^2+^ ions or L-histidine. Chem. Commun.

[16] Pelossof G., Tel-Vered R., Liu X.Q., Willner I. (2011). Amplified surface plasmon resonance based DNA biosensors, aptasensors, and Hg^2+^ sensors using hemin/g-quadruplexes and Au nanoparticles. Chem.-Eur. J.

[17] Chen J.H., Zhang J., Guo Y., Li J., Fu F.F., Yang H.H., Chen G.N. (2011). An ultrasensitive electrochemical biosensor for detection of DNA species related to oral cancer based on nuclease-assisted target recycling and amplification of DNAzyme. Chem. Commun.

[18] Mir M., Vreeke M., Katakis I. (2006). Different strategies to develop an electrochemical thrombin aptasensor. Electrochem. Commun.

[19] Lin Z.Z., Chen Y., Li X.H., Fang W.H. (2011). Pb^2+^ induced DNA conformational switch from hairpin to G-quadruplex: Electrochemical detection of Pb^2+^. Analyst.

[20] Choi C.L., Alivisatos A.P. (2010). From artificial atoms to nanocrystal molecules: Preparation and properties of more complex nanostructures. Annu. Rev. Phys. Chem.

[21] Sapsford K.E., Pons T., Medintz I.L., Mattoussi H. (2006). Biosensing with luminescent semiconductor quantum dots. Sensors.

[22] Medintz I.L., Uyeda H.T., Goldman E.R., Mattoussi H. (2005). Quantum dot bioconjugates for imaging, labelling and sensing. Nat. Mater.

[23] Gill R., Zayats M., Willner I. (2008). Semiconductor quantum dots for bioanalysis. Angew. Chem. Int. Ed.

[24] Bruchez M., Moronne M., Gin P., Weiss S., Alivisatos A.P. (1998). Semiconductor nanocrystals as fluorescent biological labels. Science.

[25] Sharon E., Freeman R., Willner I. (2010). CdSe/ZnS quantum dots-g-quadruplex/hemin hybrids as optical DNA sensors and aptasensors. Anal. Chem.

[26] Sharon E., Freeman R., Riskin M., Gil N., Tzfati Y., Willner I. (2010). Optical, electrical and surface plasmon resonance methods for detecting telomerase activity. Anal. Chem.

[27] Freeman R., Liu X.Q., Winner I. (2011). Chemiluminescent and Chemiluminescence Resonance Energy Transfer (CRET) detection of DNA, metal ions, and aptamer-substrate complexes using hemin/g-quadruplexes and CdSe/ZnS quantum dots. J. Am. Chem. Soc.

[28] Liu X.Q., Freeman R., Golub E., Willner I. (2011). Chemiluminescence and Chemiluminescence Resonance Energy Transfer (CRET) aptamer sensors using catalytic hemin/g-quadruplexes. ACS Nano.

